# A Novel Method for Classification of Running Fatigue Using Change-Point Segmentation

**DOI:** 10.3390/s19214729

**Published:** 2019-10-31

**Authors:** Taha Khan, Lina E. Lundgren, Eric Järpe, M. Charlotte Olsson, Pelle Viberg

**Affiliations:** 1 Centre of Artificial Intelligence, School of information technology, Halmstad University, SE-301 18 Halmstad, Sweden; Lina.Lundgren@hh.se (L.E.L.); eric.jarpe@hh.se (E.J.); 2 Rydberg Laboratory of Applied Science, School of business, engineering and science, Halmstad University, SE-301 18 Halmstad, Sweden; charlotte.olsson@hh.se; 3 Raytelligence AB, 302 42 Halmstad, Sweden; pelle@swedishadrenaline.com

**Keywords:** surface-electromyography, blood lactate concentration, random forest, running, fatigue

## Abstract

Blood lactate accumulation is a crucial fatigue indicator during sports training. Previous studies have predicted cycling fatigue using surface-electromyography (sEMG) to non-invasively estimate lactate concentration in blood. This study used sEMG to predict muscle fatigue while running and proposes a novel method for the automatic classification of running fatigue based on sEMG. Data were acquired from 12 runners during an incremental treadmill running-test using sEMG sensors placed on the vastus-lateralis, vastus-medialis, biceps-femoris, semitendinosus, and gastrocnemius muscles of the right and left legs. Blood lactate samples of each runner were collected every two minutes during the test. A change-point segmentation algorithm labeled each sample with a class of fatigue level as (1) aerobic, (2) anaerobic, or (3) recovery. Three separate random forest models were trained to classify fatigue using 36 frequency, 51 time-domain, and 36 time-event sEMG features. The models were optimized using a forward sequential feature elimination algorithm. Results showed that the random forest trained using distributive power frequency of the sEMG signal of the vastus-lateralis muscle alone could classify fatigue with high accuracy. Importantly for this feature, group-mean ranks were significantly different (*p* < 0.01) between fatigue classes. Findings support using this model for monitoring fatigue levels during running.

## 1. Introduction

Both recreational runners and running athletes can enhance their training outcomes by monitoring their fatigue level while training. The concept of muscular fatigue is usually described in two levels, or thresholds, where the first threshold represents neuromuscular fatigue and the second represents fatigue due to an imbalance between lactate production and lactate removal [1]. Most markers that are used to indicate fatigue during running, i.e., lactate or ventilatory parameters, need to be measured via blood samples or ventilation. However, due to the inconvenience of this during outdoor and recreational activities, neither of these are feasible to use in everyday training. Therefore, both recreational and competitive runners could be assisted by a new monitoring system that can predict fatigue level while running using noninvasive, portable instruments. Examples of such measurement units are electromyography devices, which in recent years have become small enough to be portable and possible to use inside clothing materials [2].

Previous studies have shown that features from an electromyography (EMG) signal can be used to detect fatigue thresholds both during cycling and running [3,4]. The features that have most frequently been used to calculate the fatigue thresholds are amplitude-based measures, such as the root mean square and integrated EMG, and frequency-based measures, such as mean power frequency or median frequency [3]. However, there are also studies that report a lack of direct correlation between the mean power frequency and fatigue level [5]. The validations of the above features are mainly based on comparing the time or workload of the thresholds in comparison to known critical fatigue thresholds, such as the ventilatory threshold, the neuromuscular threshold, or the lactate threshold (LT). However, few studies report methods to continuously monitor fatigue level in relation to lactate level or ventilatory oxygen uptake, to use as input into an intelligent system. One of the early attempts of an autonomous fatigue prediction system [6] used linear discriminant analysis to predict fatigue in a static exercise. Another study was successful in finding fatigue predicting features in a dynamic biceps curl exercise [7]. However, rarely are sporting activities static and localized to one muscle only, which is why vigorous activities, such as bicycling and running, are of interest to study further. 

Razanskas et al. [8] developed an algorithm using the cumulative sum of scores and correlation methods to train a machine-learning model to detect changes in lactate levels during bicycling. They found that a random forest (RF) model could predict the lactate level based on surface-electromyography (sEMG) signals with R^2^ values > 0.9 when trained using the time domain features and >0.8 when trained using the frequency domain features. However, for a model to be valid to use in a product application, it needs to be useful also for other types of activities, such as running. Furthermore, the model needs to be able to predict an individual’s fatigue level without knowledge of the workload or the state of recovery. Furthermore, LT assessed by the absolute accumulation of lactate is a subjective variable [9,10], which may provide errors in determining the current fatigue state just by finding the lactate prediction level. Therefore, it may be of interest to further explore whether better prediction accuracy can be obtained using an a priori classification model that determines the fatigue level based on lactate slope parameters. 

The purpose of this study was to investigate whether the machine learning method that was previously shown useful in detecting fatigue in bicycling using sEMG on lower limbs [8] can also predict fatigue in a running task. Furthermore, the study aimed to develop this method further by adding a segmentation of fatigue levels to the analysis in order to improve accuracy.

## 2. Materials and Methods

**Figure 1:**
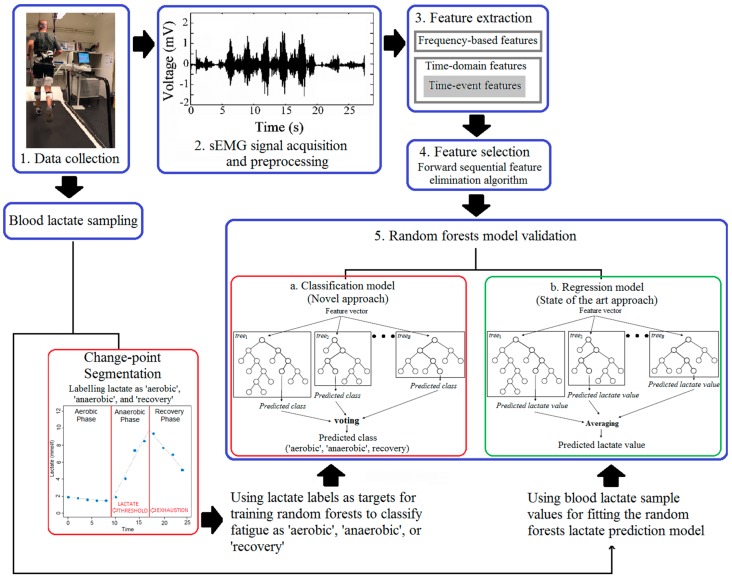
Schematic diagram of the study design. After data collection and data preprocessing, the study tested two ways to predict lactate levels of the athletes during the incremental running test, i.e., a novel fatigue classification model based on change-point segmentation and a previously described regression model for predicting lactate accumulation [8].

The overall study design is described in a schematic diagram in Figure 1. In the first step, data were acquired using a treadmill protocol, as described below, and sEMG sensors placed on the lower limb muscles of runners. Secondly, the sEMG signals were preprocessed. Three different types of feature sets were extracted from the signals, and a forward sequential feature elimination algorithm was used for feature selection. Two different RF models were trained, of which the first model was used to predict lactate accumulation using regression, as described previously by Razanskas et al. [8], and the second model to classify fatigue based on change-point segmentation of lactate accumulation. The algorithm steps are described further below.

### 2.1. Data Collection

Twelve aerobically trained participants (five women and seven men) volunteered to participate in the study. Data were acquired during a single test session of an incremental treadmill-running test, as described below. Participants were either amateur long-distance runners (n = 4) or amateur triathlon athletes (n = 8) with a mean (±SD) running volume of 46.2 (±15.6) km/week, age 43 (±8) years, body mass 71.9 (±11.7) kg, and stature 1.75 (±0.99) m. All participants were informed about the study prior to the test occasion, both orally and in writing. Ethical approval was obtained from the regional ethical review board (Reg. No. 2014/162).

**Figure 2:**
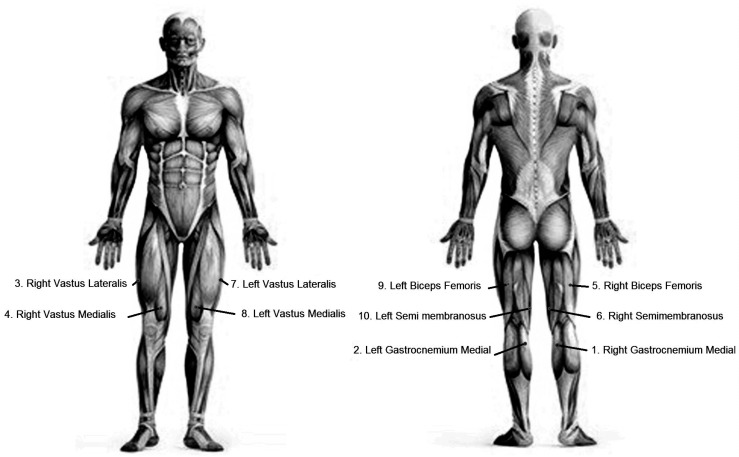
The sensor positions used for surface electromyography.

Participants were equipped with wireless sEMG-sensors (Delsys Trigno, Delsys, Boston, MA, USA) on m. Vastus Lateralis (VL), m. Vastus Medialis (VM), m. Biceps femoris (BF), m. Semitendinosus (SM), and the medial head of m. Gastrocnemius (GM), as shown in Figure 2, abbreviated for the right leg as RVL, RVM, RBF, RSM, and RGM, respectively, and for the left leg as LVL, LVM, LBF, LSM, and LGM, respectively.

The sensor positions were palpated according to the SENIAM (Surface Electromyography for the Non-Invasive Assessment of Muscles) guidelines, and sites were shaved, rubbed, and cleaned before attachment of the sensors. The sensors were attached using double-sided tape (Trigno Sensor Skin Interface, Delsys, Boston, MA, USA), and elastic sports tape around the limb to keep the sensors securely fixed. Maximum voluntary isometric contractions were performed for each muscle, according to the SENIAM guidelines, using manual resistance. Ventilatory measurements were performed using Oxycon Pro (Jaeger, Hoechberg, Germany), attached to a mask (7450 Series V2 Mask, Hans Rudolph Inc. Shawnee, Kansas, USA) worn by the participants to collect the ventilatory gases for analysis of ventilation, oxygen, and carbon dioxide. A heart rate monitor (Polar FT4, Kempele, Finland) was positioned on the thorax and connected to the Oxycon wirelessly. Subjective measures of leg fatigue and ventilatory fatigue were obtained using the Borg scale rating of perceived exertion, and blood lactate concentration samples (Lactate Pro 2, Arkray, Japan), were collected every two minutes throughout the test, by stepping to the side of the treadmill. This procedure took about 30 s. The blood samples were gathered from the right-hand fingertips of the participant. Prior to testing, each participant familiarized themselves with the perceived rating scale, the strategy to step aside from the treadmill to take a lactate sample, and the safety procedure when fatigue occurred. These procedures were practiced to enable safe and efficient data collection. 

The incremental treadmill protocol started with a light warm-up at 8 (for women) or 9 (for men) km/h. The test started with six minutes of running at a speed corresponding to just below each athlete’s 10 km tempo, which was between 11 and 13 km/h. After the initial six minutes, an increase in workload (1–1.5 km/h) was performed every two minutes until the athlete reached a lactate level above 5 mmol/L (or was unable to increase the running workload). At this stage, the workload level was kept constant until the athlete was unable to continue (minimum six minutes). The final six minutes of the test were at the initial speed, i.e., their individual 10 km tempo.

### 2.2. Feature Extraction

The sEMG signals were recorded from the muscles at a sampling rate of 1926 Hz. In order to eliminate electronic noise and motion artifacts, the sampled signals were preprocessed using the suggested Butterworth filter [11] with 10th order 400 Hz low pass filter at a stopband of 450 Hz with 60 dB attenuation and the 10th order 20 Hz high pass filter at a stopband of 10 Hz with 60 dB attenuation. The filtered signal S(t) was interpolated using Hermite cubic splines and used for feature extraction and analysis.

#### 2.2.1. Frequency Domain Features

In order to extract frequency domain features, the preprocessed signal S(t) was segmented into muscle activity bursts S_i_(t) using a method for detecting muscle activity previously described by Razanskas et al. [8]. An i^th^ muscle activity burst corresponds to a single stride cycle in the running. The power spectrum P_i_(f) of an i^th^ burst was computed by using discrete Fourier transform on S_i_(t) using Equation (1):

(1) $$P_{i}\left(f\right)=\left|\mathrm{DFT}\left(S_{i}\right)\right|.$$

P_i_(f) was further used to compute the power distribution of frequencies D_i_(f) of each i^th^ burst using Equation (2):

(2) $$D_{i}\left(f\right)=\frac{P_{i}\left(f\right)}{\int_{0}^{f_{\mathrm{Nyq}}}P_{i}\left(f\right)\mathrm{df}},$$

where f_Nyq_ is the Nyquist frequency. Frequency-based features were extracted using S_i_(t) and D_i_(f). The description of each feature is given in Table 1. Details can be found elsewhere [12].

#### 2.2.2. Time-Domain Features

The muscle activation time is a critical time-domain feature that is defined as the time difference between the activation and deactivation of a muscle during a stride. The activation moment A(i) and the deactivation moment D(i) of a muscle were computed using the local maxima and the local minima of the derivative of the sEMG signal S(t), as described by Razanskas et al. [12]. The time span of a single stride cycle was estimated using two consecutive muscle activation moments, A(i) and A(i+1). It was observed that muscles BF, VM, and VL fired sequentially during a stride cycle, i.e., the activation moment of BF preceded the activation moment of VM, and the activation moment of VM preceded the activation moment of VL. Hence, the length of time between A(i+1) and A(i) of muscle BF corresponds to the time span of one stride cycle, and the activation of other muscles can be computed as fractions of this baseline time length.

We used the activation moment A(i) of RBF as the starting timestamp because it was the first activation signal to start each activation cycle. It is pertinent that Razanskas et al. [8,12] used three sEMG channels recorded from rectus femoris, vastus lateralis, and semitendinosus muscles of both legs for estimating bicycling fatigue. In order to develop a similar model for estimating running fatigue, we used three sEMG channels recorded from BF, VM, and VL, and discarded SM and GM when extracting features based on the time domain analysis. 

The phase shift Ø_X, Y_ between the activation moments of two muscles, say X and Y, in one stride cycle, was computed using Equation (3):

(3) $$\emptyset_{X,\;Y}\left(i\right)=\;\frac{A_{Y}\left(i\right)-A_{x}\left(i\right)}{A_{\mathrm{RBF}}\left(i+1\right)-A_{\mathrm{RBF}}\left(i\right)}.$$

Similarly, the active time percentage α_X_ of muscle X in one stride cycle was computed using Equation (4):

(4) $$\alpha_{X}\left(i\right)=\;\frac{D_{x}\left(i\right)-A_{x}\left(i\right)}{A_{\mathrm{RBF}}\left(i+1\right)-A_{\mathrm{RBF}}\left(i\right)}.$$

The root-mean-square ρ_X_ of the sEMG signal S(t) of muscle X for the i^th^ cycle was computed using Equation (5):

(5) $${\;\rho }_{X}\left(i\right)=\;\frac{1}{D_{x}\left(i\right)-A_{x}\left(i\right)}.$$

Furthermore, the arithmetic mean (AM) and standard deviation (SD) of features Ø_X, Y_, α_X,_ and ρ_X_ were computed using Equations (6) and (7), respectively:

(6) $$AM=\frac{1}{n}\sum_{i=1}^{n}a_{i},$$

(7) $$SD=\sqrt{\frac{1}{n-1}\sum_{i=1}^{n}{\left(a_{i}-\bar{a}\right)}^{2}},$$

where *a_i_* is the feature value for an *i*^th^ stride cycle, and *n* is the total stride cycles. Moreover, the asymmetry of each feature was measured by computing the corresponding arithmetic means of that feature from the right and the left leg and finding the absolute difference between them, given as:

(8) $$\sum \left(*\right)=\left|Mean_{Right}\left(*\right)-Mean_{left}\left(*\right)\right|.$$

In order to reduce the model complexity, Razanskas et al. [8] used a subset by excluding amplitude-based features from the time domain features. The subset was termed as the time event features. The time-domain and time event features (shaded grey) are listed in Table 2. 

### 2.3. Feature Selection

In order to reduce the complexity of feature space as well as computational time in estimating blood lactate using sEMG, and more importantly, to identify the most relevant features for the model construction, a forward sequential feature elimination algorithm based on the Spearman correlation coefficient was used. Importantly, Razanskas et al. [8] used this feature selection method previously for predicting cycling fatigue.

As a preprocessing step, the blood lactate values were interpolated using Hermite cubic splines with Catmull–Rom tangents [13] so as to equate the number of lactate samples to the number of sEMG segments. Then, the feature values and blood lactate values were normalized between 0 and 1. Spearman rank correlation coefficient *r_s_* was computed for samples *i = 1…n* using Equation (9), given as:

(9) $$r_{s}=1-\;\frac{6\sum_{i=1}^{n}{\left(x_{i}-y_{i}\right)}^{2}}{n\left(n^{2}-1\right)},$$

where *n* is the total number of sEMG segments, *x_i_* is a feature value of an *i*^th^ segment, and *y_i_* is the blood lactate value for that segment, measured as mmol/L. The value of *r_s_* is 1 if the relationship between *x* and *y* is monotonically increasing, and –1 if the relationship between *x* and *y* is monotonically decreasing. In the first round, feature sets were selected by setting the threshold of *r_s_* as 0, i.e., all features were selected for the model construction. The threshold was increased by 0.05 in each iteration until the value was equal to 1, where the most significant and perfectly correlated features were selected for the model construction. The results are discussed in Section 3.2.

### 2.4. Random Forest

A prediction model based on RF [14] operates by constructing multiple decision trees that are trained using random subsets of the training dataset. This step of splitting the training set into random subsets and using these subsets for training the decision trees is termed bagging or bootstrap aggregation. Bagging splits the data in a way that two thirds of the random samples from the total dataset are used for training each decision tree and the remaining one third of the samples, termed out-of-bag (OOB) samples, are used for testing that tree. An error $Y_{i}-{\hat{Y}}_{i}$ is estimated for each OOB sample *i*, where *Y_i_* is the actual value of the sample and ${\hat{Y}}_{i}$ is the predicted value of the sample produced by a decision tree in the forest. 

In the case of a regression problem, the generalization performance of an RF model is estimated using an average of the coefficients of determination (R^2^), computed using Equation (10):

(10) $$R^{2}=1-\frac{\sum_{i}{\left(Y_{i}-{\hat{Y}}_{i}\right)}^{2}}{\sum_{i}{\left(Y_{i}-E\left[Y\right]\right)}^{2}},$$

where *E*[*Y*] is the mean OOB sample value, an R^2^ value of 1 signifies complete regression, and 0 signifies naïve regression. In the case of a classification problem, voting is performed between the decision trees, each predicting an output class for a particular OOB sample *i*. The class with the majority of votes in the forest, is selected as the predicted class. In both cases, regression and classification, the utilization of multiple decision trees in an RF eliminate the prediction bias that could possibly be introduced in an output of a single decision tree. Another advantage of using bagging is that the data variance is reduced, which prevents overfitting [15].

Previous studies have shown that lactate accumulation during running is highly individual [9,10], which may lead to the high variability of lactate prediction during this activity. Therefore, another method was tested alongside that proposed by Razanskas et al. [12] to incorporate subjective trends. We incorporated subjective trends of lactate concentration based on some a priori information of physiological behavior to train an RF model for predicting running fatigue. This was done by using a change-point segmentation (CPS) method, which identified timestamps at which the lactate values reached aerobic, anaerobic, and the maximum lactate accumulation level at exhaustion during the exercise. As a result, the previous regression problem to predict blood lactate accumulation in bicycling was transformed into a new classification problem that enabled the generalization of the model for predicting blood lactate accumulation in the running.

### 2.5. Change-Point Segmentation

**Figure 3:**
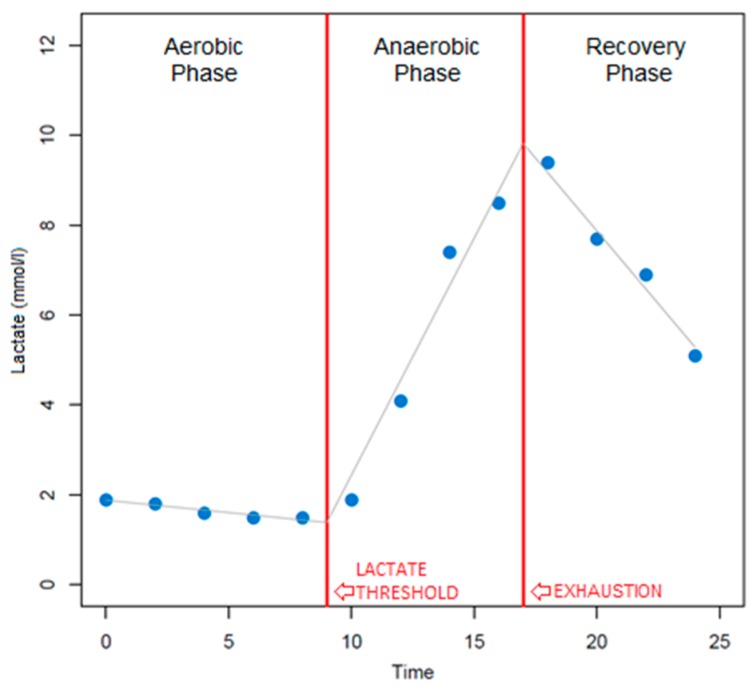
Change-point segmentation of lactate accumulation during a 25 min running session.

The change-point method segmented the duration of the total exercise time between three classes (Figure 3), based on the individual slope changes between lactate points. Class ‘1’ represented the aerobic phase, i.e., the time between the start of the test and the LT. Class ‘2’ represented the anaerobic or lactate accumulation phase, i.e., the time between the LT and the point where the lactate accumulation was maximum at exhaustion. Class ‘3’ represented the post-exhaustion lactate recovery phase, i.e., the time between the maximum lactate accumulation and during the recovery.

In order to find the best fitting segmentation of classes 1, 2, and 3, a linear piecewise connected function was defined and selected by means of the least square method. That is, given the lactate samples *x* (*t*) observed at the time points *t*_1_, *t*_2_, *t*_3_ … *t*_n_ where 0 ≤ *t*_1_ < *t*_2_ < *t*_3_ < … < *t*_n_ ≤ max, the segmentation was the pair of breaks was defined as:

(11) $$\left(t^{\prime },t^{\prime \prime }\right)={\mathrm{argmin}}_{a_{1,}b_{1,}b_{2,}b_{3,}t^{\prime },t^{\prime \prime }}\sum_{i=1}^{n}{\left(l\left(t_{i}\right)-x\left(t_{i}\right)\right)}^{2},$$

where *l*(*t*_1_), *l*(*t*_2_), *l*(*t*_3_), …, *l*(*t*_n_) are values of the piecewise connected lines, given as:

(12) $$l\left(t\right)=\left\{\begin{array}{ll} a_{1}t+b_{1} & for\;0\leq t\leq t^{\prime }\\ a_{2}\left(b_{2}\right)t+b_{2} & for\;t^{\prime }\leq t\leq t^{\prime \prime }\\ a_{3}\left(b_{3}\right)t+b_{3} & for\;t^{\prime \prime }\leq t\leq max \end{array}\right\},$$

for:

(13) $$a_{2}\left(b_{2}\right)=a_{1}+\frac{1}{t^{\prime }}\left(b_{1}-b_{2}\right),$$

and:

(14) $$a_{3}\left(b_{3}\right)=a_{2}+\frac{1}{t^{\prime \prime }}\left(b_{2}-b_{3}\right),$$

which guarantees the connection of the lines. Here, *t’* refers to the timestamp at LT and *t’’* represents the timestamp at maximum lactate accumulation at exhaustion. From this minimization procedure, class 1 is the time duration between timestamps 0 and *t’*, class 2 is between timestamps *t’* and *t’’*, and class 3 is between *t’’* and max. The recorded sEMG signals were segmented using the timestamps *t’* and *t’’* between the three classes, aerobic, anaerobic, and recovery phases, as shown in Figure 3, and used as training targets. Each lactate classification model was checked visually to ensure that the classification model performed according to other methods of determining LT [9].

### 2.6. Model Validation

Two different experiments were performed, first to validate the previous bicycling models on the running data. The second experiment was to validate the novel CPS method for the classification of running fatigue. In order to optimize the generalization performance, the forward sequential feature elimination algorithm was used in both experiments. In the first experiment, RF regression models were trained separately using the frequency-based features (Table 1), the time-domain features (Table 2), and the time event features (Table 2: shaded) extracted from the sEMG signals recorded during the running exercise. As previously suggested [8,12], RF with 100 trees was used with an initial 10 random seeds. Models were trained separately for predicting lactate as well as oxygen uptake. A comparative analysis of the RF regression models for bicycling and running is presented in Section 3.1.

In the second experiment, the new CPS-based RF classification model was trained to discriminate between running fatigue classes using the feature sets. The model was stratified using 100 trees and 10 initial random seeds. Two different approaches were used. In the first approach, full feature sets were used for training. In the second approach, the forward sequential feature elimination algorithm was used to identify the combination of features producing the highest classification accuracies. The classification performance was analyzed using the area under the receiver operating characteristic (ROC) curves (AUC). Results and comparisons between frequency, time event, and the time-domain models for the classification of running fatigue are presented in Section 3.2. Significant features that contributed to model training were examined using a non-parametric Kruskal Wallis (KW) test by grouping samples from all participants into classes 1, 2, and 3. This was done to ensure that the classification accuracy obtained by the model was not due to noise or overfitting. Importantly, a significant *p*-value (*p* < 0.05) rejected the null hypothesis that samples belong to the same group. 

## 3. Results

### 3.1. Experiment 1: Validation of the Bicycling Model on the Running Dataset

**Figure 4:**
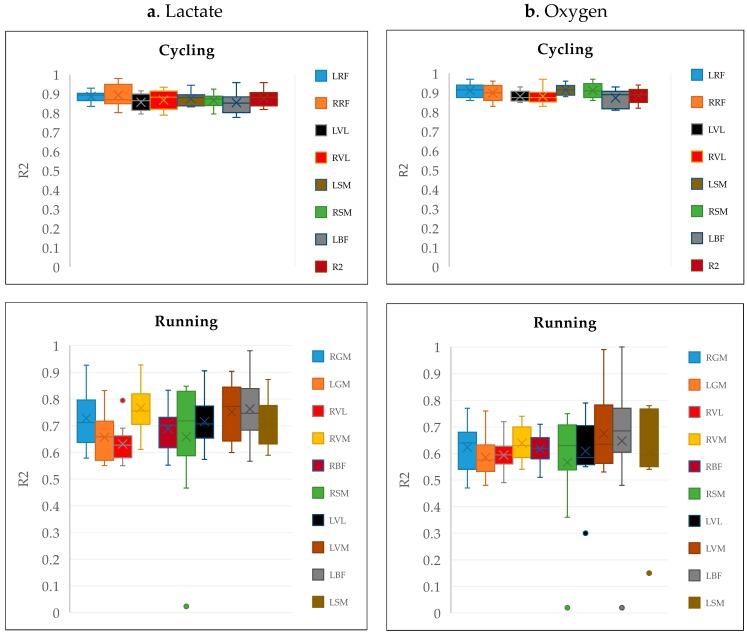
A comparison between R^2^ boxplots obtained using random forest (RF) regression and frequency-based features from cycling and running data. (**a**) depicts the boxplots estimated using lactate. (**b**) depicts the boxplots estimated using the oxygen uptake. Dots represent outliers, and crosses represent mean R^2^. Muscle abbreviations used in the cycling study: *RF: Rectus Femoris, *VL: Vastus Lateralis, *SM: Semitendinosus, *BF: Biceps Femoris. R* and L* represent right and left legs, respectively.

When using the frequency-based feature set to train the RF regression model, the results showed a low correspondence between the model performances for cycling and running, i.e., with only a few occasions of R^2^ > 80% for running (Figure 4). For the twelve runners, the best fatigue predictions based on lactate were achieved using the sensors at RVM and LVM. On average, the RF regression produced a low mean R^2^ of 0.71 ± 0.12 standard deviation for all participants compared to a robust mean R^2^ of 0.87 ± 0.045 in bicycling. Similarly, when predicting fatigue based on the oxygen uptake, the RF regression produced a low mean R^2^ of 0.62 ± 0.13 compared to a robust mean R^2^ of 0.9 ± 0.04 in bicycling.

**Figure 5:**
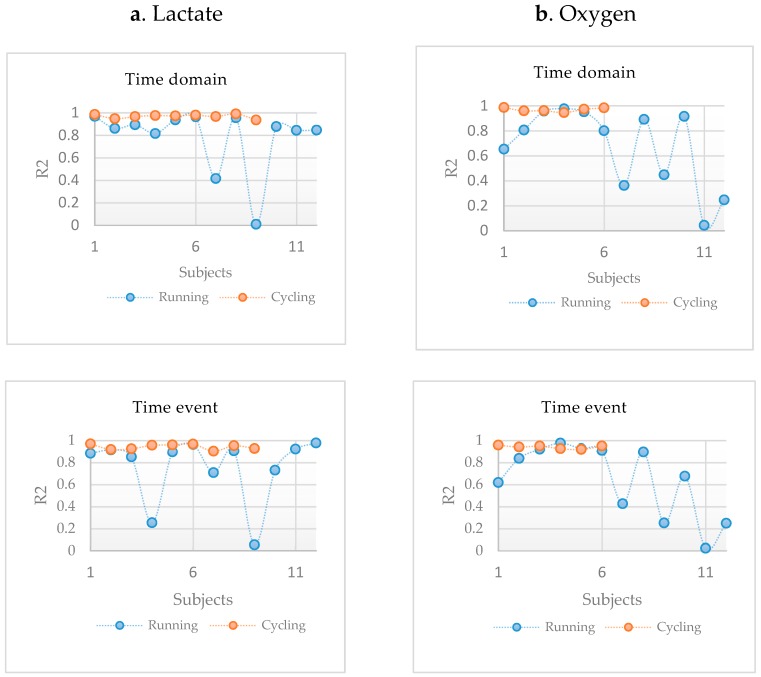
A comparison between R^2^ estimates obtained using RF regression based on the time domain and time event features from cycling and running data. (**a**) depicts R^2^ estimates for lactate. (**b**) depicts R^2^ estimates for oxygen uptake.

When using the time-event features, the model showed corresponding results to cycling on an individual basis, where 8 out of the 12 running participants showed robust estimates between the model and lactate measures (Figure 5a). The mean R^2^ in the running was 0.75 ± 0.29, compared to the mean R^2^ of 0.94 ± 0.02 in cycling. The estimations of oxygen uptake were less robust for most participants, i.e., the mean R^2^ in the running was 0.64 ± 0.32, comparatively less than the mean R^2^ of 0.94 ± 0.01 in bicycling.

Similar observations were made using the time domain features, although with 10 out of 12 participants showing robust estimations of the model in the running study (Figure 5a). Using lactate, the mean R^2^ in the running was 0.78 ± 0.28, compared to the mean R^2^ of 0.97 ± 0.01 in cycling. For the oxygen uptake, the mean R^2^ in the running was 0.67 ± 0.31, compared to the mean R^2^ of 0.96 ± 0.01 in cycling.

### 3.2. Experiment 2: The Novel Change-Point Segmentation Method for the Classification of Running Fatigue

**Figure 6:**
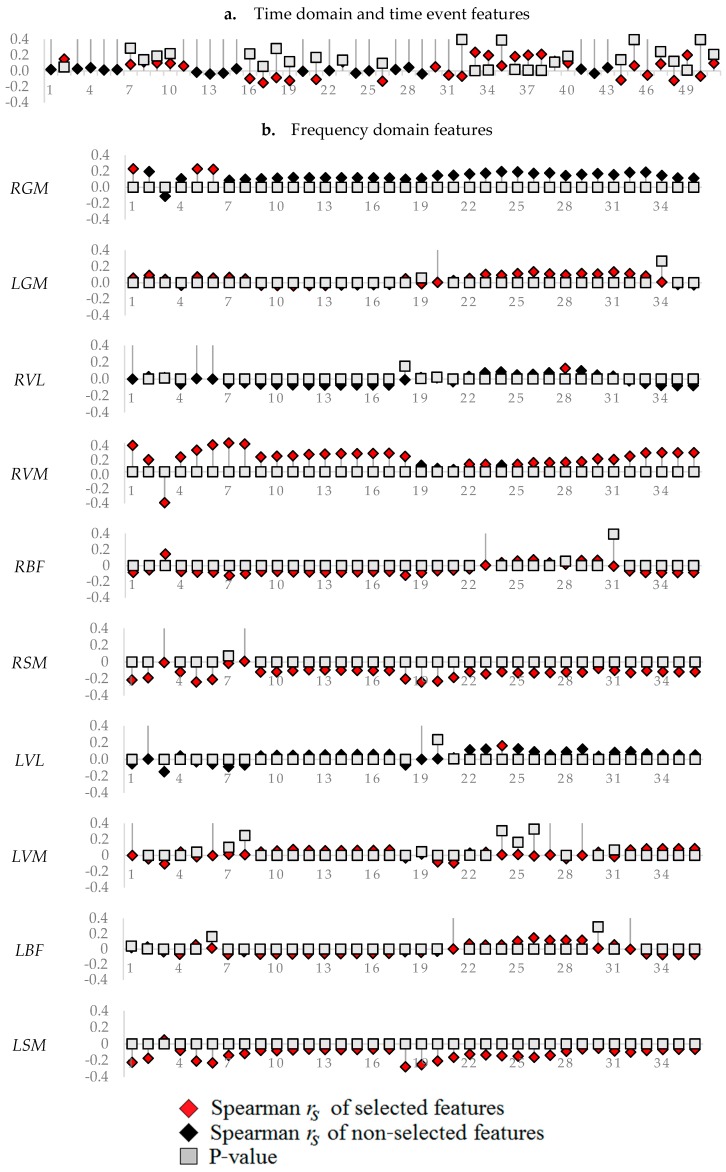
Spearman correlation between features and classes of running fatigue.

Spearman correlation between features and CPS-based fatigue classes are shown in Figure 6. Thirty-two time-domain features (2, 7–11, 16–19, 21, 23, 26, 30–40, 44–51) produced *r_s_* distinct from zero (*r_s_* ≥ 0.05), out of which seven features (2, 33, 34, 36, 37, 38, and 49) showed significant correlation with fatigue classes (*p* < 0.05). In the case of frequency-based features, 34 RGM (1–6, 8–17, 19–34), 8 LGM (23, 25–27, 29–32), 1 RVL (28), 32 RVM (1–18, 22, 23, 25–36), 4 RBF (3, 7, 8, and 18), 29 RSM (1, 2, 4–6, 9–11, 15–29, 31–36), 6 LVL (3, 22–25, and 29), 1 LVM (3), 5 LBF (25–29), and 16 LSM (1, 2, 5–8, 18–27) features produced *r_s_* significantly distinct from zero (*r_s_* ≥ 0.1; *p* < 0.05). Notably, in the cycling study, the same features correlated with lactate accumulation with average *r_s_* < 0.2 (*p* < 0.05) [8].

Selected features that contributed to maximum model performances are illustrated using red squares in Figure 6. The time event features showed the highest model performance (AUC = 0.87) using all the features in the model (Table 3a). However, when optimizing the feature set to a minimum number, then the frequency-based model for RVL and LVL performed best (AUC = 0.86) using only one feature (28 and 24, respectively) (Table 3b). In general, the proposed CPS-based classification model showed a high level of accuracy for most analyses.

**Figure 7:**
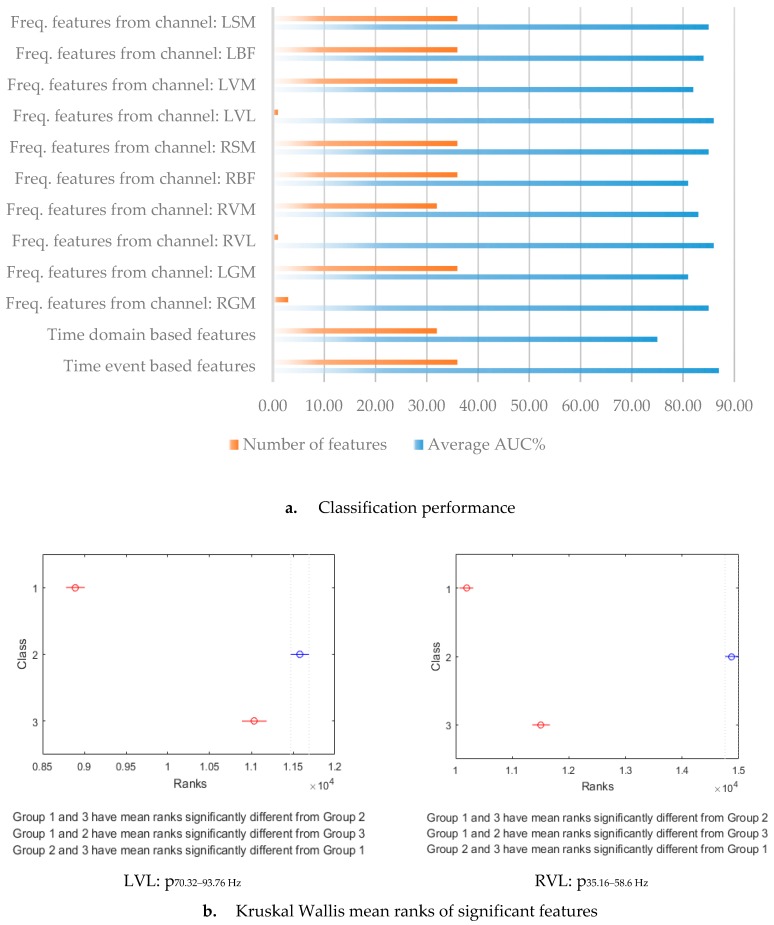
Classification performance using the change-point segmentation approach. (**a**) shows that the best AUCs of 86% were produced by two channels, RVL using feature p_35.16__–58.6 Hz_, and LVL using feature p_70.32__–93.76 Hz_. (**b**) shows the mean rank distribution of these features between fatigue classes.

Significant features of RVL and LVL models, p_35.16__–58.6 Hz_ and p_70.32__–93.76 Hz_, were further investigated. The KW test was used to examine the distribution of feature values between classes based on mean ranks (Figure 7b). In the case of RVL, significant differences were observed in the mean ranks of p_35.16__–58.6 Hz_ between the three classes at a 95% confidence level (*p* < 0.05). The mean ranks of the feature in class 2 were the highest compared to the mean ranks in classes 1 and 3. The mean ranks of the feature in class 3 were higher than the mean ranks in class 1, suggesting that the recovery phase and the aerobic phase could be discriminated significantly (*p* < 0.05) using this RVL feature.

Similarly, in the case of LVL, the mean ranks of feature p_70.32__–93.76 Hz_ were significantly different between the three classes, with a 95% confidence interval (*p* < 0.05). The mean ranks of the feature in class 2 were the highest compared to classes 1 and 3. In addition, the mean ranks of the feature in class 2 were lower than the mean ranks in class 2 but higher than the mean ranks in class 1, suggesting that the recovery phase could be discriminated significantly (*p* < 0.05) using the LVL feature. Importantly, the classification performance of the RVL feature p_35.16__–58.6 Hz_ was approximately equal to the performance of the LVL feature p_70.32__–93.76 Hz_ (Table 3b and Figure 7). Hence, considering the leg dominance in individuals, it would be desirable to use both RVL and LVL features for running fatigue classification.

## 4. Discussion

The results suggest that the method shown useful to predict fatigue in bicycling [8,12] needs development to be valid for use in running. Razanskas et al. [8,12] used RF regression models with 100 trees for predicting bicycling fatigue. Strong R^2^ values (>0.8) were produced when frequency-based features (Figure 4) were used to train the model, and the performance was improved (R^2^ > 0.9) when the time domain features were used. However, these results relied on the assumption of the cyclic stationarity of the extracted sEMG segments [16]. When we used this model to predict lactate concentration in running fatigue, a higher standard deviation in the R^2^ for different runners was observed compared to the standard deviation in the R^2^ for bikers (Figure 4), supporting that LT during running is a subjective trait [9,10]. It was observed that runners produced subjective physiological trends relative to the exercise intensity, i.e., the duration and the values of the observed lactate concentrations at aerobic and anaerobic thresholds, and the maximum lactate concentration at exhaustion was varying for different runners, making it complicated for an RF regression model to generalize the lactate accumulation trends. In addition, some runners do not attain their maximum lactate concentration. The CPS-method, however, which was developed in this study, improved model accuracy (Figure 7). The best feature found was frequency percentage features for the VL muscles. We obtained an area under the ROC above 0.8, which can be considered promising for the intended use (Table 3b). However, there may be further improvements available to increase the validity of fatigue prediction. 

Although this study showed that the model suggested previously by Razanskas et al. [8,12] did not seem valid for running, the technique using CPS showed promising results for use in general fatigue prediction. However, it needs to be validated for several types of endurance activities, such as running and bicycling outdoors. A successful outcome of validation studies may result in sensor implementation into clothing that can be used to keep track of lactate accumulation without collecting blood samples during training.

Previous studies that have determined thresholds (aerobic and anaerobic threshold) based on lactate measures from incremental exercise tests used a number of different methods to perform this, for example, fixed levels, increments of 0.5–1.5 mmol/L, and various slope gradients [9]. The method used to segment the LT in this study was based on a combination of previous methods and CPS methods [9]. The piecewise linear function estimates the slopes of the first two segments and put a threshold (segment breakpoint) where the two lines intersect, similar to the approach of Bunc et al. [17]. There are some issues regarding the validity of the LT based on incremental exercise tests, and which method works best seems to depend on the individual [18]. However, the segmentation approach in this study was mainly aimed to map and estimate feature prediction from the EMG-data, which seemed to work well as a first step. 

Something to take into consideration when developing this method further is that the LT may occur at a lower exertion level in cycling compared to running [19]. Therefore, a future study may be recommended to test the method on both running and cycling using the same athletes and setup, and involve more ecologically valid contexts, such as varied terrain.

Although this study aimed to compare the results with the previously performed study by Razanskas et al. [12], there were some methodological differences, due to the type of exercise performed. Firstly, the exercise protocol differed in building up to the highest workload. The running protocol made incremental increases up to the maximum level, whereas the cycling study went from 60% of maximum to 90% of maximum directly. The reason for incremental increases during running was for the central circulatory system to be able to adapt to the new workload before the increase in workload. Furthermore, by increasing from 60% to 90% without incremental steps, there will be an instantaneous effect of workload above the LT, which makes it more challenging to determine the transition between a completely aerobic phase and an anaerobic phase. 

The practical application of this study implies that machine learning techniques of EMG-signals can be used to predict whether the running athlete is currently in a phase where there is a balance between lactate production and lactate removal (aerobic state), or whether lactate is accumulating in the blood (anaerobic state). Although the results of this study seem promising, in use, the algorithm will require calibration at an individual level to be valid. This is due to the individual differences in LT and maximal fatigue capacity. A calibration procedure could be performed as a treadmill run with incremental steps whilst giving feedback on a rate of perceived effort scale. Although most users of a fatigue-monitoring product would likely be experienced runners and cyclists, it may be considered a risk to promote running to exhaustion, which is why a submaximal procedure would be preferable. 

It is essential in a practical application that as few features as possible are used to optimize the estimates of the outcome, due to signal processing time [20]. In this study, the two features that showed the best estimate of the segmentation model for running was the relative power of the signal (p_35.16–58.6 Hz_ of the RVL and p_70.32–93.76 Hz_ of the LVL muscle). Therefore, the recommendation would be to involve these features in a minimized setting of fatigue analysis during running. 

In conclusion, the proposed CPS-based classification algorithm was able to generalize the discrimination between lactate accumulation phases for different participants that were previously not possible using the bicycling model. The method is an alternative to the previous lactate estimation from blood samples that were not feasible for use in everyday training. Our experiments have shown that only one feature based on the power spectrum of the sEMG signal could estimate fatigue levels and the state of recovery, suggesting that sEMG signals could be processed accurately in real-time as a result of the lower time and computational complexity of the model. The algorithm was able to generalize the given population. However, we plan to validate the new model further to test ecological validity.

## Figures and Tables

**Table 1:** Frequency-based features extracted from an i^th^ segment of the surface-electromyography (sEMG) signal S_i_(t) obtained from each muscle.

Number	Name	Description
1	RMS	Root mean square error of S_i_(t).
2	dRMS	The backward difference of the root mean square error of S_i_(t).
3	IF	Instantaneous frequency or zero-crossings of S_i_(t) divided by two.
4	ModF	Mode of D_i_(f).
5	MnF	Mean of D_i_(f).
6	StD	The standard deviation of D_i_(f).
7	Skew	The skewness of D_i_(f).
8	Kurt	Kurtosis of D_i_(f).
9–17	q_0.1_ to q_0.9_	Every 10th percentile of D_i_(f), i.e., q_0.5_ is the 50th percentile or the median power frequency.
18–36	p_23-47 Hz_ to p_234-258 Hz_	Relative power contained in 23.44 Hz bands (width of 6 DFT bins) of power spectrum P_i_(f) with an overlap of 11.72 Hz, starting with a band of 23.44–46.88 Hz and ending with a band of 234.4–257.8 Hz.

**Table 2:** Time domain and time event features (shaded grey) of sEMG signals. Two numbers assigned to a feature correspond to the arithmetic mean and standard deviation of that feature.

	Number	Symbol	Number	Symbol
Time event features	The mean and standard deviation of phase shifts $\emptyset_{X,\;Y}$ between X and Y muscles for total strides		The mean and standard deviation of the signal root-mean-square $\rho_{X}$ of muscle X for total strides	
	1,2	$\;\emptyset_{\mathrm{RBF},\;\mathrm{RVM}}\;$	31, 32	$\;\rho_{\mathrm{RBF}}\;$
	3,4	$\;\emptyset_{\mathrm{RBF},\;\mathrm{RVL}}\;$	33, 34	$\;\rho_{\mathrm{RVM}}\;$
	5,6	$\;\emptyset_{\mathrm{RVM},\;\mathrm{RVL}}\;$	35, 36	$\;\rho_{\mathrm{RVL}}\;$
	7,8	$\;\emptyset_{\mathrm{LBF},\;\mathrm{LVM}}\;$	37, 38	$\;\rho_{\mathrm{LBF}}\;$
	9,10	$\;\emptyset_{\mathrm{LBF},\;\mathrm{LVL}}\;$	39, 40	$\;\rho_{\mathrm{LVM}}\;$
	11,12	$\;\emptyset_{\mathrm{LVM},\;\mathrm{LVL}}\;$	41, 42	$\;\rho_{\mathrm{LVL}}\;$
	13,14	$\;\emptyset_{\mathrm{RBF},\mathrm{LBF}}\;$	Asymmetry of $\emptyset_{X,\;Y}$ between the right and left leg.	
	15,16	$\;\emptyset_{\mathrm{RVM},\;\mathrm{LVM}}\;$	43	$\;\sum_{\left(\emptyset_{\mathrm{BF},\mathrm{VM}}\right)}\;$
	17,18	$\;\emptyset_{\mathrm{RVL},\;\mathrm{LVL}}\;$	44	$\;\sum_{\left(\emptyset_{\mathrm{BF},\;\mathrm{VL}}\right)}\;$
	The mean and standard deviation of the active time percentages $\alpha_{X}$ of muscle X for total strides		45	$\;\sum_{\left(\emptyset_{\mathrm{VM},\;\mathrm{VL}}\right)}\;$
	19,20	$\;\alpha_{\mathrm{RBF}}\;$	Asymmetry of $\alpha_{X}$ between the right and left leg.	
	21,22	$\;\alpha_{\mathrm{RVM}}\;$	46	$\;\sum_{\alpha_{\mathrm{BF}\;}}\;$
	23, 24	$\;\alpha_{\mathrm{RVL}}\;$	47	$\;\sum_{\alpha_{\mathrm{VM}\;}}\;$
	25, 26	$\;\alpha_{\mathrm{LBF}}\;$	48	$\;\sum_{\alpha_{\mathrm{VL}\;}}\;$
	27, 28	$\;\alpha_{\mathrm{LVM}}\;$	Asymmetry of $\rho_{X}$ between the right and left leg.	
	29, 30	$\;\alpha_{\mathrm{LVL}}\;$	49	$\;\sum_{\rho_{\mathrm{BF}\;}}\;$
Total number of time-domain features = 51 Total number of time event features = 36			50	$\;\sum_{\rho_{\mathrm{VM}\;}}\;$
			51	$\;\sum_{\rho_{\mathrm{VL}\;}}\;$

**Table 3:** A comparison between the classification performances based on the area under the ROC curves (AUC) using the time-event, time-domain, and frequency-based features. Classes 1, 2, and 3 represent aerobic, anaerobic, and recovery phases, respectively. (**a**) lists AUCs produced using full feature sets. (**b**) lists AUCs produced using the selected features.

**a.** Using all Features					**b.** Using Selected Features					
Model	AUC				AUC				Features	
	Class 1	Class 2	Class 3	Average	Class 1	Class 2	Class 3	Average	No. of Selected Features	Selected Features
Time-domain models										
*Time event*	0.90	0.84	0.87	0.87	0.90	0.84	0.87	0.87	36	All
*Time domain*	0.77	0.76	0.71	0.74	0.76	0.75	0.74	0.75	32	2, 7–11, 16–19, 21, 23, 26, 30–40, 44–51
Frequency-domain models										
*RGM*	0.82	0.82	0.75	0.80	0.86	0.86	0.82	0.85	3	1st, 5th, and 6th
*LGM*	0.80	0.83	0.79	0.81	0.80	0.83	0.79	0.81	36	All
*RVL*	0.75	0.78	0.69	0.74	0.86	0.87	0.86	0.86	1	28th
*RVM*	0.86	0.84	0.79	0.83	0.86	0.84	0.80	0.83	32	1–18, 22, 23, 25–36
*RBF*	0.82	0.84	0.77	0.81	0.82	0.84	0.77	0.81	36	All
*RSM*	0.85	0.87	0.84	0.85	0.85	0.87	0.84	0.85	36	All
*LVL*	0.83	0.82	0.74	0.80	0.86	0.86	0.85	0.86	1	24th
*LVM*	0.84	0.86	0.78	0.82	0.84	0.86	0.78	0.82	36	All
*LBF*	0.85	0.86	0.80	0.84	0.85	0.86	0.80	0.84	36	All
*LSM*	0.83	0.87	0.86	0.85	0.83	0.87	0.86	0.85	36	All
*Average*	0.82	0.84	0.78	0.82	0.84	0.86	0.82	0.84		
